# Supplementary material for: Isoschaftoside in Fig Leaf Tea Alleviates Nonalcoholic Fatty Liver Disease in Mice via the Regulation of Macrophage Polarity
Source: Nutrients. 2025 Feb 21;17(5):757. doi: 10.3390/nu17050757 (PMC11902273; doi:10.3390/nu17050757)
Supplement: Supplementary file 1 [file nutrients-17-00757-s001.zip › sup Data S1.docx]

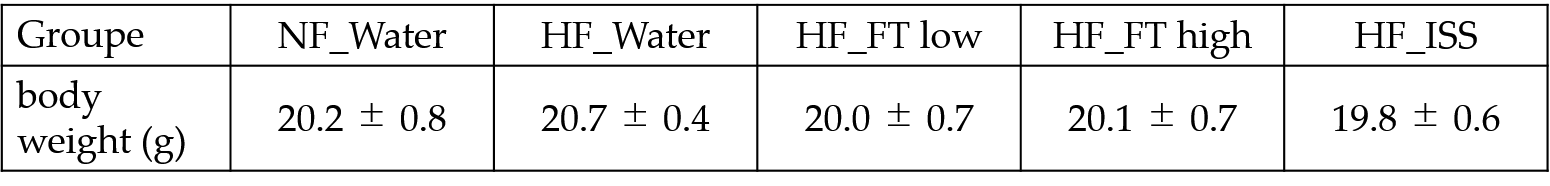


The results are means ± SD, with n = 6 per group.

**Supplementary Data S1.** Mean body weight at time of grouping.
