# Supplementary material for: Isoschaftoside in Fig Leaf Tea Alleviates Nonalcoholic Fatty Liver Disease in Mice via the Regulation of Macrophage Polarity
Source: Nutrients. 2025 Feb 21;17(5):757. doi: 10.3390/nu17050757 (PMC11902273; doi:10.3390/nu17050757)
Supplement: Supplementary file 1 [file nutrients-17-00757-s001.zip › sup Data S2.docx]

Data S2

**Supplementary Data S2.** The blood levels of ALP and insulin at 17 weeks. Blood samples were taken from the vena cava. The results are means ± SD, with n ≥ 5 per group.
