# Supplementary material for: Isoschaftoside in Fig Leaf Tea Alleviates Nonalcoholic Fatty Liver Disease in Mice via the Regulation of Macrophage Polarity
Source: Nutrients. 2025 Feb 21;17(5):757. doi: 10.3390/nu17050757 (PMC11902273; doi:10.3390/nu17050757)
Supplement: Supplementary file 1 [file nutrients-17-00757-s001.zip › sup Data S3.docx]

**Supplementary Data S3.** Gene expression of HF induced NAFLD groups.

| **Pathways of NAFLD** | **Probe Name** | **Annotation** | | **Relative expression**  **(vs. NF_water)** | | |
| --- | --- | --- | --- | --- | --- | --- |
|  |  | **Gene**  **Symbol** | **GeneName** | **HF_water** | **HF_FT low** | **HF_FT high** |
| **Lipid metabolism** | A_52_P257774 | Cyp4a10 | cytochrome P450, family 4, subfamily a, polypeptide 10 | -4.17 | -1.45 | -2.87 |
|  | A_51_P401921 | Srebf1 | sterol regulatory element binding transcription factor 1 | 2.01 | 2.79 | 2.92 |
| **oxidative stress** | A_55_P2082733 | Cybb | cytochrome b-245, beta polypeptide | 10.25 | 3.29 | 5.44 |
|  | A_55_P1979341 | Cyba | cytochrome b-245, alpha polypeptide | 6.30 | 2.64 | 3.89 |
|  | A_51_P207031 | Ncf1 | neutrophil cytosolic factor 1 | 3.83 | 1.52 | 2.29 |
| **fibrogenesis** | A_52_P525107 | Col1a1 | collagen, type I, alpha 1 | 7.16 | 1.67 | 3.03 |
|  | A_51_P182303 | Col1a2 | collagen, type I, alpha 2 | 4.37 | 1.54 | 2.42 |
|  | A_51_P167527 | Lum | lumican | 2.22 | 1.36 | 1.35 |
|  | A_52_P87713 | Timp1 | tissue inhibitor of metalloproteinase 1 | 2.87 | 1.33 | 1.60 |
|  | A_51_P390715 | Tgfb1 | transforming growth factor, beta 1 | 2.21 | 1.39 | 1.84 |
